# In Slico Screening and In Vitro Identification of Hyperuricemia-Inhibiting Peptides from *Trachurus japonicus*

**DOI:** 10.3390/foods14030524

**Published:** 2025-02-06

**Authors:** Zexuan Xu, Miaoyu Gan, Weiliang Guan, Fang Tian, Yuxi Wang, Jinjie Zhang, Luyun Cai

**Affiliations:** 1 College of Food and Pharmaceutical Sciences, Ningbo University, Ningbo 315211, China; 2211390081@nbu.edu.cn; 2 Ningbo Innovation Center, College of Biosystems and Food Science, Zhejiang University, Ningbo 315100, China; ganmy2021@163.com (M.G.); f265a519@163.com (Y.W.); 3 School of Light Industry and Food Engineering, Guangxi University, Nanning 530004, China; wlguan@gxu.edu.cn; 4 Key Laboratory of Health Risk Factors for Seafood of Zhejiang Province, School of Food and Pharmacy, Zhejiang Ocean University, Zhoushan 316022, China; tianfang@zjou.edu.cn

**Keywords:** molecular docking, molecular dynamics, peptides, hyperuricemia, uric acid

## Abstract

Hyperuricemia arises from imbalanced uric acid metabolism, contributing to gout and related chronic diseases. When traditional drugs are used to treat hyperuricemia, side effects are inevitable, which promotes the exploration of new bioactive compounds. Protein hydrolysates and peptides are gradually showing potential in the treatment of hyperuricemia. This study investigated the uric acid inhibitory activity of peptides extracted from *Trachurus japonicus* using in silico and in vitro methods. We employed in silico virtual enzymolysis and experimental validation to identify bioactive peptides from *Trachurus japonicus* proteins. Four peptides (DF, AGF, QPSF, and AGDDAPR) were comprehensively screened by molecular docking and database analysis. After solid-phase synthesis, the inhibitory effects of these peptides on hyperuricemia were further verified in vitro and at the cellular level. The results showed that all four peptides have good hyperuricemia-inhibiting activities. Molecular docking and molecular dynamics revealed that peptides DF and AGDDAPR affect the production of uric acid by binding to the active sites of urate transporter 1 (URAT1), glucose transporter 9 (GLUT9), and xanthine oxidase (XOD), while peptides QPSF and AGF mainly influence the XOD active site, confirming that it is feasible to rapidly screen hyperuricemia-inhibiting peptides by molecular docking.

## 1. Introduction

The escalating incidence of gout and hyperuricemia worldwide, attributed to changes in dietary habits and lifestyle, has become a critical issue in public health. According to the National Health and Nutrition Examination Survey, the prevalence of gout and hyperuricemia in the United States stands at 3.9% and 21.4%, respectively, with a significant disparity in rates between genders, where men are affected at a rate of 5.2%, substantially higher than the 2.7% observed in women [1]. This condition is associated with the consumption of purines and the concentration of uric acid (UA) in the blood, which is the final metabolite of purine breakdown. Hyperuricemia is diagnosed when serum UA levels exceed 360 μmol/L in women and 420 μmol/L in men [2]. It is a risk factor for a range of chronic health issues, including renal failure, hypertension, obesity, diabetes, and cardiovascular diseases, as well as metabolic syndrome [3,4,5]. The management of asymptomatic hyperuricemia, which lacks the clinical signs of gout or kidney disease, is a subject of ongoing debate [6]. Existing therapeutic options, such as allopurinol and febuxostat, while effective, are not without risks, particularly the potential for serious adverse reactions like allopurinol hypersensitivity syndrome (AHS) [7,8]. Despite existing therapies, there is a need for safer alternatives derived from natural sources, which this study aims to address.

Compared to conventional medications, many food-derived bioactive peptides are generally considered safe, with advantages such as low toxicity, ease of absorption, and high specificity when appropriately processed and administered. Aquatic products are a rich source of these underexplored biological peptides. The extraction of bioactive peptides from marine sources holds substantial market potential, particularly in the food industry. *Trachurus japonicus* is a widely available marine fish with a relatively high annual yield. During the processing of this fish, a significant number of by-products such as minced meat are generated. These by-products are currently of low economic value and are often underutilized [9]. Therefore, they are considered a potential raw material for further utilization, such as the extraction of bioactive peptides. As natural derivatives in end products, these active substances exhibit antihypertensive [10], antioxidant [11], immunomodulatory [12], antibacterial [13], and anticancer effects [14], playing a crucial role in the development of nutritional supplements and functional foods.

Traditional enzymatic hydrolysis has been a cornerstone in the processing of bioactive peptides, yet it is not without its drawbacks. This method can be labor-intensive and generates a significant amount of waste, which is complex and time-consuming to deal with. In contrast to conventional enzymatic hydrolyzing methods, contemporary computational approaches such as ExPASy PeptideCutter, PeptideRanker, and SwissADME offer an efficient, accurate, and cost-effective means to anticipate and analyze the release and characteristics of bioactive peptides [15]. Molecular docking technology enables the screening of active peptides from nearly fully hydrolyzed proteins and predicts their interactions with target molecules, such as receptor proteins [16]. This approach can uncover the intrinsic mechanisms of interaction between peptides and receptor proteins, providing a theoretical foundation for the development of new bioactive substances. Computational chemistry analysis further enhances our understanding and the application potential of these peptides, rendering the research process more efficient and cost-effective. The aim of this research is to acquire anti-UA peptides by means of the in silico virtual enzymolysis of *Trachurus japonicus* protein, validate them via solid-phase synthesis and in vitro assays, and investigate the interactions between bioactive peptides and receptor proteins through molecular docking and molecular dynamics techniques.

## 2. Materials and Methods

### 2.1. Materials

Xanthione oxidase (XOD) from milk, 1,1-Diphenyl-2-picrylhydrazyl (DPPH), 2,2′-azino-bis(3-ethylbenzothiazoline-6-sulfonic acid) (ABTS), a xanthine oxidase assay kit, and glutathione (GSH) were purchased from Solarbio Co., Ltd. (Beijing, China). Allopurinol was purchased from Aladdin Co. Ltd. (Shanghai, China). UA was purchased from Sigma-Aldrich Co. LLC. (St. Louis, MO, USA). A Cell Counting Kit-8 (CCK-8) was obtained from APExBIO Technology LLC. (Houston, TX, USA). Other chemicals were provided by Sinopharm Chemical Reagent Co., Ltd. (Shanghai, China) and were of analytical grade.

### 2.2. In Silico Hydrolysis of Trachurus japonicus Protein

The amino acid sequences pertaining to *Trachurus japonicus* proteins were retrieved from the NCBI database, including myoglobin (NCBI: BAH90799.1), β-actin (NCBI: AEV42295.1), parvalbumin (NCBI: AEW90939.1), and obscurin-parvalbumin (NCBI: BAE46763.1). The processes by which these proteins are digested by pepsin, trypsin, and chymotrypsin A were simulated using the ExPASy PeptideCutter tool [17]. The absorption efficiency of peptides depends on the length of their chains. Shorter peptide chains are associated with enhanced absorption by the human body [18]. Therefore, peptides with seven or fewer amino acids and a molecular weight of less than 1 kDa were selected, and peptide segments with a bioactivity score higher than 0.4 were screened out by PeptideRanker. The toxicity of these peptide segments was predicted using ToxinPred. Finally, the SwissADME platform was used to evaluate the absorption, distribution, metabolism, excretion, and toxicity (ADMET) characteristics of the selected peptide segments, as well as to analyze their pharmacokinetic parameters, including their water solubility, to comprehensively evaluate the bioactivity, safety, and pharmacokinetic properties of these short peptides.

### 2.3. Virtual Screening Based on Molecular Docking

#### 2.3.1. Construct Receptor Proteins

Bovine XOD retains all the catalytically essential residues of the human enzyme, with an overall sequence homology of over 90% [19], and was therefore chosen for the screening of XOD inhibitors via molecular docking [20,21,22]. The urate transporter 1 (URAT1) protein is a key protein for urate reabsorption among renal transporters, and the glucose transporter 9 (GLUT9) protein is the sole transporter responsible for the efflux of urate from cells into the blood among renal transporters. The structures of the bovine XOD-quercetin complex (PDB: 3NVY), URAT1 (PDB: 9B1L) [23], and GLUT9 (PDB: 8Y65) [24] were obtained from the Protein Data Bank (PDB) (https://www.rcsb.org) [25]. Unnecessary ligand molecules and water molecules were removed using AutoDock Tools software (ADT, 1.5.7) [26]. Treatments such as adding polar hydrogen atoms and Gasteiger charges were performed, followed by exporting PDBQT files. The GetBoxplugin (https://github.com/MengwuXiao/GetBox-PyMOL-plugin, accessed on 25 June 2023) was utilized to design the center coordinates of the molecular docking boxes, which were (28.02, 14.09, 108.04), (100.01, 93.03, 98.06), and (20.06, 0.50, −9.12), respectively. The cavity size was set to 30 Å × 30 Å × 30 Å, the energy range was 16, the exhaustiveness was 32, and the number of modes was 20. These docking boxes covered all the amino acid residues of the active sites of XOD, URAT1, and GLUT9. The optimal model was selected based on energy and binding patterns and used to explore the interactions and reported drugs and peptides were used as positive controls.

#### 2.3.2. Construct Three-Dimensional Peptide Structure Ligands

The PRPM program (https://github.com/RazzyChen/PRPM, accessed on 25 June 2023), which was developed based on Py-Rosetta [27], was employed to build and optimize the three-dimensional configurations of the peptides. The initial construction of the three-dimensional structures of the peptides was carried out in accordance with the approach described by Shen et al. [28]. After that, ADT was introduced to append polar hydrogen atoms and Gasteiger charges, as well as to conduct flexibility processing on the small-molecule ligands. Subsequently, the files were exported in the PDBQT format.

#### 2.3.3. Molecular Docking

Molecular docking was performed by utilizing the AutoDock Vina software suite (ADV, 1.2.5). For the visualization of the interactions between ligands and receptors after docking, the open-source PyMol (2.1.0) software and the free visualization module of the Discovery Studio 2024 client were utilized to perform visualization processing on the data.

### 2.4. Solid-Phase Peptide Synthesis (SPPS)

Based on the results of the virtual screening, the peptides DF, AGF, QPSF, and AGDDAPR, with a purity of 99%, were synthesized by solid-phase peptide synthesis at Sangon Biotech (Shanghai) Co., Ltd. (Shanghai, China).

### 2.5. In Vitro Assays of XOD Inhibitory and Antioxidant Capabilities

#### 2.5.1. XOD Inhibitory Assay

The in vitro XOD inhibitory assay was carried out by adopting the approach of Zhao et al. [29], with minor alterations. Xanthine and XOD were, respectively, dissolved in phosphate-buffered solution (PBS, with a pH value of 7.4) at concentrations of 1.5 mM and 0.1 U/mL. Peptides of different concentrations (10, 20, 40, 80, and 160 mM) were dissolved in the PBS. A total of 120 μL of the sample solution, 150 μL of PBS, and 100 μL of XOD solution were combined and allowed to react thoroughly at 37 °C for 15 min. Subsequently, 120 μL of xanthine solution was added and the reaction was continued for an additional 20 min. Eventually, 150 μL of 1 M HCl was added to halt further enzymatic activity, and the absorbance was determined at 295 nm by means of an ultraviolet spectrophotometer (TU-1810PC, Purkinje, Beijing, China). The XOD inhibition rate was calculated according to Equation (1).

*XOD inhibition rate* (%) = [1 − (*A*_1_ − *A*_3_)/(*A*_2_ − *A*_4_)] × 100% (1)

where *A*_1_ is the absorbance after the mixed reaction of the inhibitor, XOD, and xanthine; *A*_2_ is the absorbance after the reaction of xanthine and XOD without adding the inhibitor; *A*_3_ is the absorbance of the sample and xanthine without adding XOD; and *A*_4_ is the absorbance of the xanthine solution without adding XOD and the inhibitor. According to the regression curve of the relationship between the XOD activity inhibition rate and the sample concentration, the inhibitory concentration (IC₅₀ value) of the active substance that generated a 50% inhibition of XOD activity was calculated.

#### 2.5.2. Antioxidant Activity Assay

The antioxidant activity assay was carried out with appropriate modifications while referring to the method of Gan et al. [30]. A total of 100 μL of sample solution was mixed with 100 μL of ethanol DPPH solution (0.1 mM) in a 96-well plate. The absorbance was measured at 517 nm after incubating in the dark at 25 °C for 30 min. In the control group, 100 μL of DPPH solution was mixed with 100 μL of absolute ethanol. The DPPH free radical scavenging rate was calculated according to Equation (2).

(2) $$\mathrm{DPPH}\;\mathrm{radical}\;\mathrm{scavenging}\;\mathrm{rate}\;(\%)=(1-\frac{A_{1}-A_{2}}{A_{0}})\;\times \;100\%$$

where *A*_0_ is the absorbance of the blank control, represented by ethanol; *A*_1_ is the absorbance of the test samples mixed with DPPH; and *A*_2_ is the absorbance of the test samples mixed with ethanol.

By referring to the method of Wang et al. [31], with suitable modifications, an ABTS stock solution was generated through mixing a 2.45 mM K_2_S_2_O_8_ solution and a 7 mM ABTS solution in equal amounts and then leaving them to stand at 25 °C for 16 h. Prior to utilization, the absorbance value at 734 nm of the ABTS stock solution was adjusted to 0.70 ± 0.02 with deionized water. A total of 3 mL of the ABTS working solution was combined with 1 mL of the test sample. The absorbance was determined at 734 nm following incubation in the dark at 25 °C for 10 min. The ABTS free radical scavenging rate was calculated according to Equation (3).

(3) $$\mathrm{ABTS}\;\mathrm{radical}\;\mathrm{scavenging}\;\mathrm{rate}\;(\%)=(1-\frac{A_{1}-A_{2}}{A_{0}})\;\times \;100\%$$

where *A*_0_ is the absorbance of the blank control, represented by deionized water; *A*_1_ is the absorbance of the test samples mixed with ABTS; and *A*_2_ is the absorbance of the test samples mixed with deionized water.

### 2.6. Cytotoxicity Assessment

HK-2 cells were procured from the National Collection of Authenticated Cell Cultures (SCSP-511, Shanghai, China). The cytotoxicity of the peptides was appraised through a CCK-8 assay in line with the methodology of Gan, Cao, Cai, Xiang, Li, and Luan [30]. HK-2 cells were inoculated in 96-well plates (3 × 10^4^ cells per well) and cultivated in Dulbecco’s Modified Eagle Medium (DMEM; provided by Life Technologies Corp., New York, NY, USA) supplemented with 10% fetal bovine serum (FBS; from Life Technologies Corp., New York, NY, USA) at 37 °C in an atmosphere containing 5% CO_2_ for a duration of 24 h. Peptides were introduced into the wells, and, following a 24 h incubation period, 10 μL of CCK-8 reagent was added to each well and incubated for 1 h. The absorbance was gauged at 450 nm using a microplate reader (Spark, Tecan, Austria).

### 2.7. Measurement of Intracellular UA

The cell modeling process was carried out by following the method of Mao et al. [32], with certain modifications. HK-2 cells were cultured in DMEM supplemented with 10% FBS at 37 °C under a 5% CO_2_ environment for 24 h. The positive group and the experimental group were, respectively, cultured with 0.1 mM allopurinol and 2 mM peptides for 24 h. The control group and the model group were also cultured in the medium for the same period of time. Subsequently, the experimental group, the positive group, and the model group were incubated with 2.5 mM adenosine for 30 h and then further incubated with XOD (at a concentration of 0.005 U/mg) for 12 h. The content and concentration of UA in the cell supernatant were measured using a XOD assay kit.

### 2.8. Western Blot Analysis

The protein expressions of URAT1 and GLUT9 within cells were detected by means of Western blot, in accordance with the method of Mao, Jiang, and Mao [32]. The primary antibodies were as follows: URAT1 (1:1000; Cat#14937-1AP; Proteintech, Wuhan, China), GLUT9 (1:1000; Cat#ab223470; Abcam, Shanghai, China), and β-actin (1:5000; Cat#ab6276; Abcam). The secondary antibodies were as follows: Goat Anti-Mouse IgG H&L (HRP) (Cat#ab205719; Abcam) and Goat Anti-Rabbit IgG H&L (HRP) (Cat#ab6721; Abcam). The protein levels were quantitatively gauged using ImageJ software (1.8.0.112).

### 2.9. Molecular Dynamics

Molecular docking uncovers the static interactions between anti-UA peptides and protein receptors. Hence, molecular dynamics should be incorporated to verify the dynamic characteristics of these bindings. Molecular dynamics simulations (MDSs) were utilized to further assess the stability of the interaction mechanisms between protein receptors and anti-UA peptides. Sobtop-1.0 (dev5) was adopted to add atomic charges and select the Gaff force field for generating ligand topology files, and SPDBV-4.10 was employed to repair the protein–ligand structures [33]. Given that the amino acid residues of the XOD active site, which generate interaction forces through molecular docking, are all located in the C chain, Pymol was utilized to delete the A and B chains of XOD so as to reduce computational resources. Then, Gromacs 2020.6 software was used to construct the amber99sb ff [34] protein force field for XOD and conduct MDSs for 100 ns. CHARMM-GUI was used to construct the structures of URAT1 and GLUT9 in the membrane environment and then Gromacs was used to conduct MDSs for 100 ns [35,36,37,38,39,40,41,42,43,44]. Anti-UA peptides were subjected to MDS for 100 ns with XOD, URAT1, and GLUT9 in Gromacs, and the results were analyzed using DuIvyProcedures (DIP, 1.0.3) software and Gromacs [45].

### 2.10. Statistical Analysis

All data are presented as the mean ± standard deviation derived from at least three independent experiments. Statistical analysis was conducted through a one-way analysis of variance (ANOVA) with the help of SPSS v.27. Duncan’s test was employed to assess the statistical differences among samples, and a *p*-value less than 0.05 was regarded as significant. Data plotting was executed using Origin 2021.

## 3. Results and Discussion

### 3.1. Virtual Enzymatic Hydrolysis of Trachurus japonicus Protein

The *Trachurus japonicus* proteins obtained from NCBI were enzymatically hydrolyzed using pepsin, trypsin, and chymotrypsin A through the ExPASy PeptideCutter tool (https://web.expasy.org/peptide_cutter/, accessed on 25 June 2023). A total of 147 peptides were obtained after computer-assisted enzymatic hydrolysis, and peptides with scores greater than 0.4, as screened by PeptideRanker (http://distilldeep.ucd.ie/PeptideRanker/, accessed on 25 June 2023), were selected for molecular docking screening.

### 3.2. Virtual Screening of Peptides

**Figure 1:**
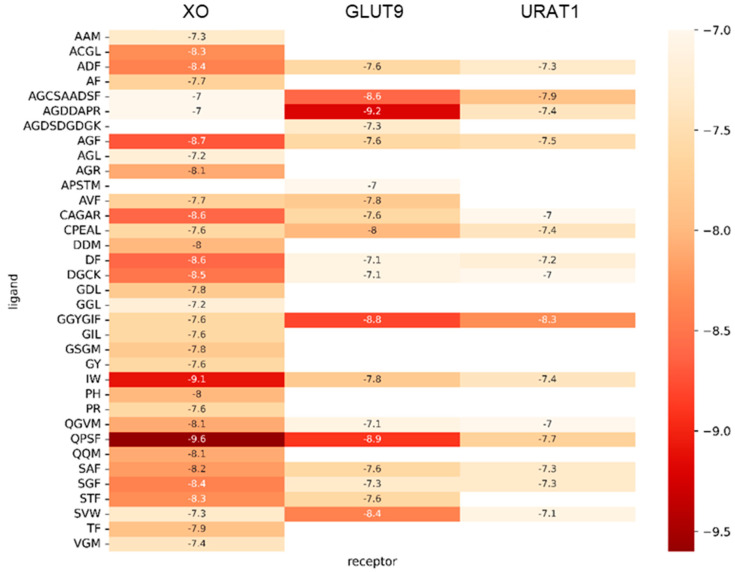
Peptides with binding energies of ≤−7.0 kcal/mol with the XOD, GLUT9, and URAT1 receptor proteins.

Virtual screening techniques mainly rely on molecular docking and databases. It is a common principle that a lower affinity score indicates a stronger binding interaction between a ligand and its receptor. The known drug allopurinol was docked with the XOD protein receptor with a binding energy of −7.3 kcal/mol. The peptide IW, which was already considered an anti-UA peptide, was docked with the XOD, URAT1, and GLUT9 receptors with binding energies of −9.1 kcal/mol, −7.8 kcal/mol, and −7.4 kcal/mol, respectively. For the convenience of further analysis, peptides with binding energies of ≤−7.0 kcal/mol with the three receptor proteins were selected as promising candidates for having anti-UA properties, as depicted in Figure 1. This threshold was chosen based on its correlation with experimentally validated inhibitory activity in prior studies. Finally, four peptides were ultimately screened out by using the SwissADME and Toixinpred servers. Combined with the analysis of the molecular weights and pharmacokinetics of various peptides in Table 1, four peptides, namely DF, AGF, QPSF, and AGDDAPR, were finally selected. They are safe and non-toxic and possess good solubility and gastrointestinal absorption.

### 3.3. In Vitro XOD Inhibitory and Antioxidant Activities of Peptides

**Figure 2:**
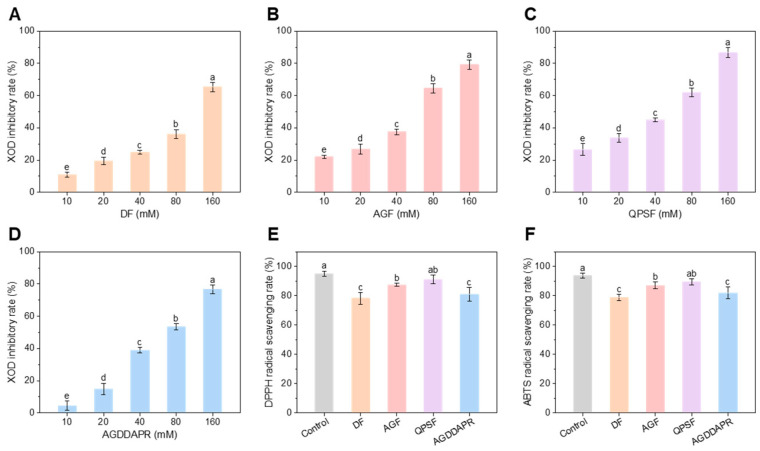
The XOD inhibitory and antioxidant activities of peptides. In vitro XOD inhibitory activities of (**A**) DF, (**B**) AGF, (**C**) QPSF, and (**D**) AGDDAPR at different concentrations. (**E**) DPPH and (**F**) ABTS scavenging rates of four peptides at a concentration of 20 mM. The control column represents the scavenging activity of glutathione. The error bars represent the standard deviation (*n* = 3). Different lowercase letters in the same column indicate a statistically significant differences between groups (*p* < 0.05).

After the solid-phase synthesis of the four screened peptides, their XOD inhibitory rates and antioxidant activities were measured. The results showed that as the peptide concentration increased, the XOD inhibition rate gradually rose (Figure 2). The IC₅₀ values of peptides DF, AGF, QPSF, and AGDDAPR for XOD were 107.64 ± 15.88 mM, 48.60 ± 9.95 mM, 38.39 ± 7.25 mM, and 65.96 ± 10.87 mM, in sequence. This indicates that these peptides, especially QPSF, exhibit desirable activities and a dose–effect relationship in terms of being anti-UA. They could regulate the generation or metabolic process of UA by inhibiting the activity of XOD. Meanwhile, in the determination of antioxidant activity, at a concentration of 20 mM, the DPPH and ABTS scavenging rates of the four peptides were consistent with the trend of their XOD inhibitory activity (Figure 2E,F). The scavenging rates for both free radicals were above 78%, suggesting that the ability of these peptides to scavenge oxidative free radicals also contributes to their inhibition of XOD activity. In both of the XOD inhibition and antioxidant activity assays, the efficacy of the peptides was ranked as follows: QPSF > AGF > AGDDAPR > DF.

### 3.4. Effects of Peptides on HK-2 Cells

**Figure 3:**
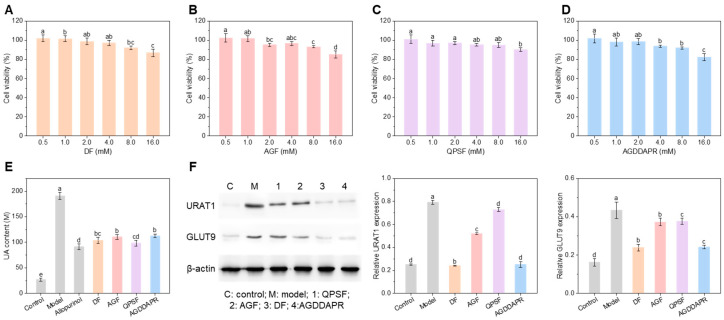
Effects of peptides on HK-2 cells. Cell viability of HK-2 cells after incubation with different concentrations of (**A**) DF, (**B**) AGF, (**C**) QPSF, and (**D**) AGDDAPR. (**E**) UA content in HK-2 cells. (**F**) URAT1 and GLUT9 protein expression levels in HK-2 cells with peptides. β-actin was used as an internal control. The error bars represent the standard deviation (*n* = 3). Different lowercase letters in the same column indicate a statistically significant differences between groups (*p* < 0.05).

For the purpose of detecting the cytotoxic impacts of peptides QPSF, AGDDAPR, DF, and AGF on HK-2 cells, the cell viability of HK-2 was examined by means of the CCK-8 method, following treatment with these peptides at varying concentrations. As depicted in Figure 3A–D, while no significant cytotoxicity was observed at concentrations up to 16 mM, slight reductions in cell viability occurred at higher doses (4–16 mM). This sub-cytotoxic effect may stem from the peptides’ short chain length, high solubility, and low hydrophobicity, which reduce nonspecific membrane interactions. Additionally, the 24 h exposure period and inherent resilience of HK-2 cells may contribute to this tolerance. Future studies will investigate lower, physiologically relevant concentrations to optimize safety and efficacy. Consequently, 4 mM of the peptides (DF, AGF, QPSF, AGDDAPR) and 0.1 mM of allopurinol were ultimately chosen for the subsequent XOD inhibitory activity experiment. 

Figure 3E shows the changes in the UA levels in HK-2 cells with hyperuricemia. The model group, treated with adenosine and XOD, exhibited significantly elevated uric acid levels, mimicking hyperuricemia. In contrast, the control group, which received no treatment, maintained normal uric acid levels. The use of allopurinol, DF, AGF, QPSF, and AGDDAPR reduced the UA levels in the cells by 52.25%, 45.57%, 42.21%, 48.61%, and 41.03%, respectively. Although the UA-lowering activities of the four peptides did not reach that of allopurinol, the control group, all four peptides at a concentration of 4 mM could achieve the similar reduction in cellular UA as 0.1 mM of allopurinol. This indicates that all four peptides can be regarded as potential health product candidates for the treatment of hyperuricemia. It has been reported that the long-term treatment of hyperuricemia with peptides is more efficient and safer than using drugs [32].

### 3.5. Molecular Docking Analysis

To illustrate the role of peptides in inhibition of XOD, the interaction forces between the enzyme and peptides were analyzed using the free visualization modules of Pymol and Discovery studio (2024 client).

#### 3.5.1. Analysis of XOD Protein

**Figure 4:**
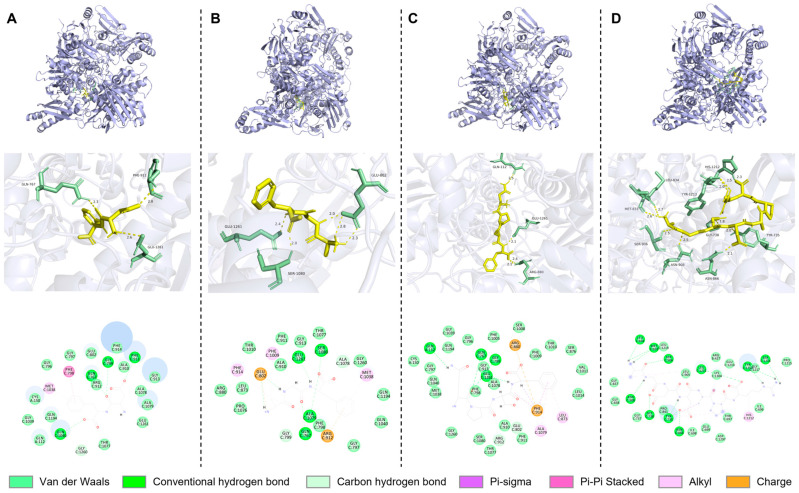
Molecular docking between peptides and XOD. Three-dimensional and two-dimensional structural diagrams of molecular docking of (**A**) DF, (**B**) AGF, (**C**) QPSF, and (**D**) AGDDAPR with XOD.

It is reported that XO is a key enzyme in purine metabolism [46]. Its molybdate domain is composed of 13 amino acid residues (G1u1261, Phe649, Thr1010, Arg880, Phe914, Phe1009, Asn768, Lys771, Val1011, Glu802, Ser876, Leu783, and Leu1014) and is its main catalytic active site [47]. Among them, the residues Glu1261, Arg880, and Glu802 are crucial for inhibiting the enzymatic activity of XO. In addition [32], some residues at the channel entrance, such as Leu648, Asn768, His875, and Phe1013, play a certain role in substrate entry [48]. The results of molecular docking, as shown in Figure 4, indicate that the inhibitory peptide can be embedded into the active center of the enzyme as the N-terminus or C-terminus, thereby preventing the binding of the substrate to the enzyme and further affecting the production of uric acid to achieve the effect of reducing the amount of uric acid present.

As shown in Figure 4A, the peptide DF forms conventional hydrogen bonds with Phe911 (2.80 Å), Gly799 (2.88 Å), Gln767 (2.31 Å), and Gln1040 (2.76 Å); forms a carbon–hydrogen bond with Gly1260 (3.69 Å); and forms Pi–Pi Stacked and Pi–Alkyl bonds with Phe798 (4.32 Å) and Met1038 (5.00 Å). The peptide DF achieves the effect of inhibiting XO activity by binding to the residues around the center of XO and does not interact with any important amino acid residues, which may be the reason for its relatively weak XO inhibitory activity compared to the other three peptides. As shown in Figure 4B, the peptide AGF forms conventional hydrogen bonds with Glu802 (2.02 Å, 2.27 Å), Glu1261 (2.41 Å, 2.45 Å), Ser1080 (2.04 Å), Ala1079 (3.02 Å), and Gln767 (2.64 Å); forms Pi–Alkyl bonds with Met1038 (5.18 Å), Phe1009 (4.66 Å), and Phe914 (3.77 Å); and engages in electrostatic interactions (Attractive Charge, Pi-Cation) with Glu802 (3.38 Å) and Arg912 (4.06 Å). The reason for the high XO inhibitory rate of the peptide AGF may be its interaction with three important amino acid residues in XO. As shown in Figure 4C, the peptide QPSF forms conventional hydrogen bonds with Gln112 (2.47 Å), Gln767 (2.99 Å), Gly799 (2.91 Å), and Glu1261 (2.09 Å, 2.14 Å); forms Pi–Sigma, Pi–Pi Stacked, Pi–Alkyl, and Anion–Pi Stacked bonds with Ala1079 (5.38 Å), Leu873 (5.41 Å), and Phe914 (4.20 Å, 2.36 Å, 4.21 Å); and engages in electrostatic interactions (Attractive Charge, Pi-Anion) with Arg880 (3.04 Å) and Phe914 (4.79 Å). The peptide QPSF interacts with three important residues in the molybdate domain and has more than one interaction with Arg880 and Phe914, which may be the reason for the high XO inhibitory activity of QPSF. As shown in Figure 4D, the peptide AGDDAPR forms conventional hydrogen bonds with Tyr735 (1.98 Å, 3.39 Å), Gly738 (1.79 Å), Asn866 (2.07 Å), Asn908 (2.48 Å), Leu834 (2.65 Å), Met833 (2.73 Å, 3.02 Å), Ser906 (2.47 Å), Leu1211 (3.29 Å), Tyr1213 (2.50 Å, 2.58 Å), and Ser1214 (2.83 Å) and forms Pi–Sigma and Pi–Alkyl bonds with His1212 (4.83 Å), Tyr735 (3.59 Å, 5.06 Å), and Leu1211 (5.36 Å). The large number of conventional hydrogen bonds may be the mechanism by which the peptide AGDDAPR inhibits XO.

It is reported that Phe914 of XO engages in hydrophobic interactions with several XO inhibitors, such as quercetin, febuxostat, allopurinol, the peptide YNVTGW, the peptide WML, and the peptide WDQW, and this force may play an important role in inhibiting XO activity [20,25,49,50,51], which may be the reason for the high XO inhibitory activity of the peptides QPSF and AGF. It is reported that having at least one charged amino acid and a hydrophobic amino acid is more conducive to antioxidant and XO inhibitory activities. The benzene ring of aromatic amino acids can participate in the formation of hydrophobic interactions, and charged residues can participate in the formation of ionic bonds. All of the above four peptides contain one or more charged amino acids and hydrophobic amino acids [52], which may also be the reason why peptides containing these amino acids exhibit stronger antioxidant and XO inhibitory activity. In addition, the in vitro XO inhibitory activity of the peptides is also consistent with the results obtained from molecular docking (Figure 4): peptide QPSF (−9.6 kcal/mol, IC_50_ = 38.392 ± 7.254 mM), peptide AGF (−8.7 kcal/mol, IC_50_ = 48.599 ± 9.947 mM), peptide DF (−8.6 kcal/mol, IC_50_ = 107.635 ± 15.881 mM), peptide AGDDAPR (−7.0 kcal/mol, IC_50_ = 65.962 ± 10.873 mM). This indicates that it is feasible to screen peptides with high XO inhibitory activity using the molecular docking method.

#### 3.5.2. Analysis of URAT1 Protein

**Figure 5:**
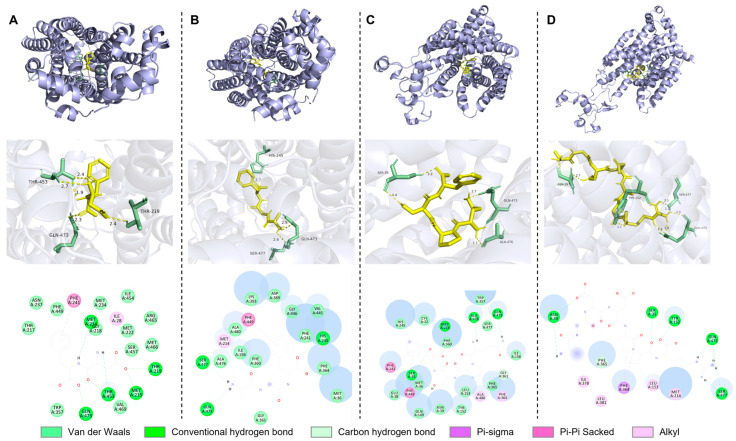
Molecular docking between peptides and URAT1. Three-dimensional and two-dimensional structural diagrams of molecular docking of (**A**) DF, (**B**) AGF, (**C**) QPSF, and (**D**) AGDDAPR with URAT1.

The URAT1 protein serves as a pivotal protein in the reabsorption of urate among renal transporters. According to relevant reports, the urate binding site within the URAT1 protein is encircled by several large aromatic residues. Specifically, five phenylalanine residues, namely Phe241, Phe360, Phe364, Phe365, and Phe449, combine to form a hydrophobic cage. This cage enwraps urate through the combined action of hydrophobic and Pi–Pi stacking interactions. In addition to these, other key amino acids, including Arg487, His245, LYS393, Met36, Asn39, Ser35, and Met214, also constitute an integral part of the protein’s structure [23]. The molecular docking results, as depicted in the accompanying Figure 5, reveal that inhibitory peptides have the ability to embed themselves into the transport center of the receptor protein, either at the N-terminus or the C-terminus. By doing so, these peptides can effectively prevent the binding of urate to the transport protein. This, in turn, impacts the production of uric acid and ultimately achieves the desired effect of reducing uric acid levels.

In Figure 5A, it can be observed that the peptide DF forms conventional hydrogen bonds with Gln473 (at a distance of 2.33 Å), Thr453 (at distances of 1.86 Å and 2.66 Å), Met215 (at 3.00 Å), Thr219 (at 2.45 Å), and Met214 (at 2.95 Å). Moreover, it engages in Pi–Pi Stacked and Pi–Alkyl interactions with Met214 (at 5.23 Å), Phe241 (at 4.96 Å), and Ile28 (at 5.33 Å). The peptide DF not only interacts with the key amino acid residue Met214 within URAT1 and its surrounding amino acids but also forms a Pi–Pi Stacked interaction with Phe241 in the hydrophobic cage of URAT1. Through these interactions, it exerts an influence on the transport of urate via URAT1. As shown in Figure 5B, the peptide AGF forms conventional hydrogen bonds with His245 (at 2.69 Å), Ser477 (at 2.64 Å), and Gln473 (at 2.75 Å). Additionally, it engages in Pi–Pi Stacked and Pi–Alkyl interactions with Phe449 (at 4.37 Å) and Met214 (at 5.08 Å). In Figure 5C, the peptide QPSF forms conventional hydrogen bonds with Ser35 (at distances of 2.23 Å and 2.40 Å), Met214 (at 3.61 Å), Ala476 (at 2.67 Å), and Gln473 (at 2.65 Å). It also forms a carbon–hydrogen bond with Gly361 (at 3.43 Å) and engages in Pi–Pi Stacked and Pi–Alkyl interactions with Phe241 (at 4.74 Å), Phe449 (at 4.99 Å), Ala480 (at 3.82 Å), and Phe364 (at 5.22 Å). Figure 5D illustrates that the peptide AGDDAPR forms conventional hydrogen bonds with Gln473 (at distances of 2.51 Å, 1.90 Å, and 2.29 Å), Asn39 (at 2.70 Å and 2.75 Å), Ser477 (at 2.10 Å and 2.53 Å), Ser35 (at 2.74 Å), and Tyr152 (at 2.13 Å). It further forms a carbon–hydrogen bond with Phe365 (at 3.66 Å) and engages in Pi–Sigma, Amide–Pi Stacked, and Pi–Alkyl interactions with Phe364 (at distances of 3.71 Å and 5.13 Å), Met214 (at 4.81 Å), Leu153 (at 4.73 Å), Leu378 (at 4.14 Å), and Leu381 (at 4.95 Å). The peptide AGDDAPR generates multiple types of interactions with the key amino acid residues of URAT1, namely Asn39, Ser35, and Met214. Furthermore, it has several Pi-related interactions with the amino acid residue Phe364 in the hydrophobic cage of URAT1. It is likely that these interaction patterns constitute the mechanism by which the peptide AGDDAPR influences the transport of urate.

#### 3.5.3. Analysis of GLUT9 Protein

**Figure 6:**
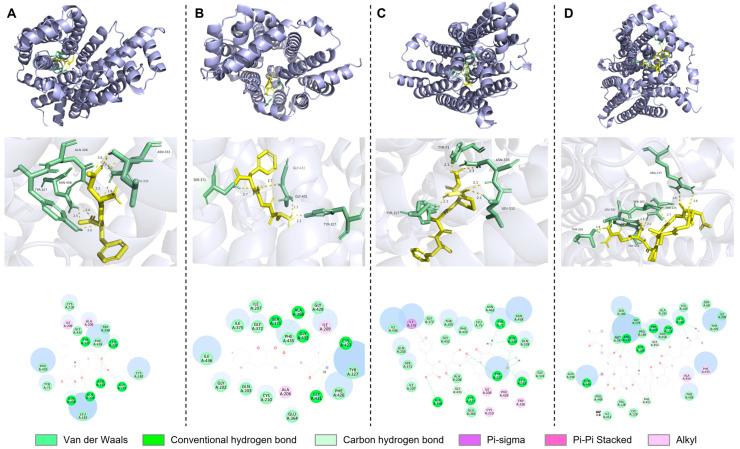
Molecular docking between peptides and GLUT9. Three-dimensional and two-dimensional structural diagrams of molecular docking of (**A**) DF, (**B**) AGF, (**C**) QPSF, and (**D**) AGDDAPR with GLUT9.

The GLUT9 protein serves as the only transporter in charge of facilitating the outflow of urate from cells into the bloodstream among the renal transporters. Nevertheless, its mechanism for transporting urate differs significantly from that of the URAT1 protein. Unlike URAT1, GLUT9 lacks a phenylalanine hydrophobic cage. The principal active site of GLUT9 consists of 14 amino acid residues, namely Trp336, Asn333, Tyr327, Leu75, Ile209, Leu332, Val213, Glu364, Cys210, Ala206, Asn462, Cys427, Asn458, Phe426, and Leu182. As depicted in Figure 6, which shows the results of the molecular docking, inhibitory peptides are capable of being inserted into the transport center of the receptor protein at either the N-terminus or the C-terminus. Through this insertion, they can effectively prevent urate from binding to the transport protein, which further impacts the production of uric acid and ultimately realizes the goal of reducing uric acid levels.

In Figure 6A, the peptide DF forms conventional hydrogen bonds with Tyr327 (at a distance of 3.06 Å), Leu332 (at 2.04 Å), Gly331 (at 2.97 Å), Asn333 (at distances of 1.87 Å and 1.89 Å), Gln328 (at 2.19 Å), and Asn458 (at 1.97 Å). Additionally, it engages in Pi–Alkyl interactions with Ile209 (at 4.50 Å) and Ala206 (at 5.22 Å). The peptide DF interacts with three key amino acid residues, and the other amino acid residues with which it interacts are also in close proximity to these key ones. This might well be the reason underlying its relatively high inhibitory activity towards GLUT9. Figure 6B illustrates that the peptide AGF forms conventional hydrogen bonds with Ser371 (at 2.66 Å), Ala368 (at 3.16 Å), Gly432 (at 2.74 Å), Cys427 (at 3.56 Å), and Gly431 (at 2.35 Å). Moreover, it engages in Pi–Alkyl interactions with Ala206 (at distances of 3.89 Å and 4.89 Å) and Ile209 (at 3.82 Å). As shown in Figure 6C, the peptide QPSF forms conventional hydrogen bonds with Ala368 (at 3.19 Å), Cy427 (at 3.05 Å), Tyr327 (at distances of 2.51 Å and 2.25 Å), Leu332 (at 2.11 Å), Asn333 (at distances of 2.06 Å and 2.30 Å), and Tyr71 (at 2.08 Å). It also forms a carbon–hydrogen bond with Gly431 (at 3.50 Å) and engages in Pi–Sigma and Pi–Alkyl interactions with Ile375 (at 3.86 Å), Cys210 (at 4.68 Å), Ile209 (at 4.96 Å), Phe426 (at 5.40 Å), and Trp336 (at 5.11 Å). In Figure 6D, the peptide AGDDAPR forms conventional hydrogen bonds with Gln446 (at 3.39 Å), Arg137 (at distances of 2.59 Å and 1.95 Å), Ser183 (at 2.67 Å), Trp459 (at 2.31 Å), Thr125 (at 2.17 Å), and Leu182 (at distances of 1.96 Å and 3.07 Å). It further forms a carbon–hydrogen bond with Phe451 (at 3.68 Å) and engages in Amide–Pi Stacked and Pi–Alkyl interactions with Phe451 (at distances of 4.52 Å, 4.31 Å, and 4.35 Å), Ala454 (at 5.05 Å), and Phe435 (at 5.49 Å). The peptide AGDDAPR interacts with key amino acid residues, and the other amino acid residues that it interacts with are also close to these key ones. This is likely the cause of its high inhibitory activity against GLUT9.

### 3.6. Molecular Dynamics Analysis

The semi-flexible molecular docking approach, which involves the docking of a flexible ligand with a rigid receptor, commonly overlooks the structural flexibility of the receptor protein. Consequently, it is essential to conduct MD simulations of the docking results in order to further validate the extent and stability of the binding interaction between a peptide and a receptor protein.

#### 3.6.1. XO Molecular Dynamics Analysis

**Figure 7:**
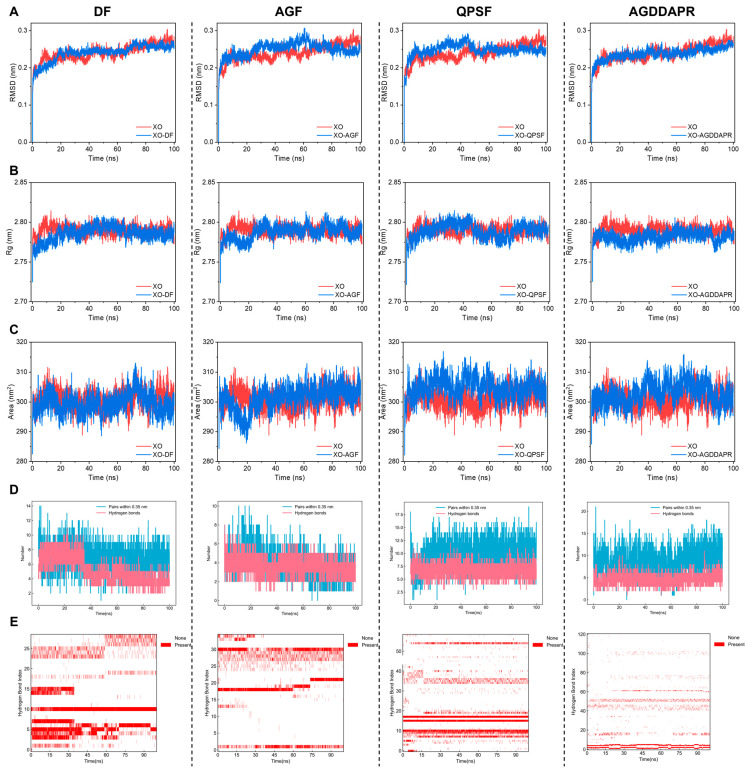
Molecular dynamics analysis of XO. (**A**) RMSD, (**B**) Rg, (**C**) SASA, (**D**) the number of hydrogen bonds, and (**E**) hydrogen bond occupancy during the molecular dynamics simulation of DF, AGF, QPSF, and AGDDAPR with XO.

Molecular dynamics (MD) simulations were employed to further elucidate the structures of the complexes formed by XO and the four peptides within the molecular docking results. Figure 7A presents the root mean square deviation (RMSD) values of XO and the peptides DF, AGF, QPSF, and AGDDAPR. RMSD analysis provides a quantitative measure of the changes in protein structure during the simulation, and the stability of the RMSD value is a crucial indicator for assessing whether the simulation has reached an equilibrium state. Generally, in a system, a dynamic equilibrium steady state is considered to be achieved when the fluctuation of the protein remains relatively constant within a range of 0.1 nm at the end of the simulation [53]. In the initial stage, from 0 to 20 ns, all four complexes and XO exhibited significant upward adjustments, which can be regarded as normal structural fine-tuning during their initial adaptation to the simulation environment. The fluctuation ranges of the XO-DF and XO-AGDDAPR complexes were consistent with that of XO, showing a slow upward trend. The XO-AGF complex had a relatively large upward fluctuation during 20–60 ns, reaching its maximum fluctuation at 60 ns, and finally attained a stable state. The XO-QPSF complex had a slow upward trend from 20 to 45 ns, followed by a relatively large downward fluctuation from 40 to 55 ns, and finally reached a stable state. The RMSD values of XO and the four complexes at 100 ns were all below 0.3 nm, and the values of the complexes were even lower, demonstrating that all reached a stable state at the end of the simulation and were more stable than the single protein. In addition, combined with the molecular docking results, and due to the stronger interaction forces generated by the peptides QPSF and AGF when occupying the active site of the enzyme compared to peptides DF and AGDDAPR, the enzyme active residues were pulled, resulting in greater conformational changes.

The radius of gyration (Rg) can describe the changes in the looseness of the protein–peptide chain, assess the rigidity of the protein backbone, and serve as an indicator for characterizing the compactness of the protein structure. The solvent-accessible surface area (SASA) is used to analyze the changes in the accessibility of the protein to the solvent and can also be used to evaluate the tightness of the peptide–receptor protein complex. Generally, a lower SASA value indicates a more compact molecular structure to the complex. As shown in Figure 7B,C, the SASA and Rg values of the four complexes were lower than those of the single XO protein during the stable simulation period, indicating that the binding of the small molecules reduced the surface area of the protein and made the protein’s molecular structure more compact, suggesting that high structural stability once again plays a positive role in enzyme inhibition. In conclusion, the above results demonstrate that the peptides DF, AGF, QPSF, and AGDDAPR can interact with the enzyme and form relatively stable complexes within 100 ns of simulation. Their structural stability contributes to enhancing the inhibitory effect of the peptides on the enzyme. Hydrogen bonds are one of the main driving forces behind the binding of peptides to receptor proteins and play a vital role in maintaining a compact and appropriate structure.

Figure 7D,E display the number and occupancy of hydrogen bonds, and Figure S1 shows the angles and distances of the hydrogen bonds with the highest occupancy, as well as the amino acid residues that form the hydrogen bonds. The peptide DF had a relatively high hydrogen bond binding rate in the first 30 ns, but after 30 ns, the number of hydrogen bonds stabilized between three and five. Among them, several hydrogen bonds that interacted with the XO active site were intermittent, which might explain the relatively low in vitro XO inhibitory activity of the peptide DF. The hydrogen bonds formed by the peptide AGF with XO were relatively stable, with the number remaining between two and five. Moreover, the number of hydrogen bonds did not change even with the decrease in the number of pairs within a range of 0.35 nm. In addition, combined with the molecular docking results and Figure S1, it was shown that the peptide AGF had multiple stable hydrogen bond bindings with the amino acid residue (Glu802) of the XO active site, and Glu802 is considered one of the three most important amino acid residues in the active site. The hydrogen bonds formed by the peptide QPSF with XO were relatively firm, with the number around 5–10. Molecular docking and the Figure S1 indicated that the peptide QPSF formed multiple stable hydrogen bonds with several amino acid residues (Gln1040, Arg880, Glu1261, Gly799, and Thr1010) of an important XO active site, which might be the reason for the high in vitro XO inhibitory activity of the peptide QPSF. The peptide AGDDAPR formed approximately 3–6 relatively stable hydrogen bonds with XO. Based on the combination of molecular docking analyses we performed, the amino acid residues it bound did not change significantly, indicating that it firmly controlled the amino acid residues near the active site, thereby inhibiting XO activity. In summary, the peptides QPSF, AGF, and DF might potentially act as XO inhibitors by occupying the substrate binding site and preventing the substrate from binding to the enzyme, while the peptide AGDDAPR becomes a potential XO inhibitor by firmly occupying the amino acid residues near the active site.

#### 3.6.2. URAT1 Molecular Dynamics Analysis

**Figure 8:**
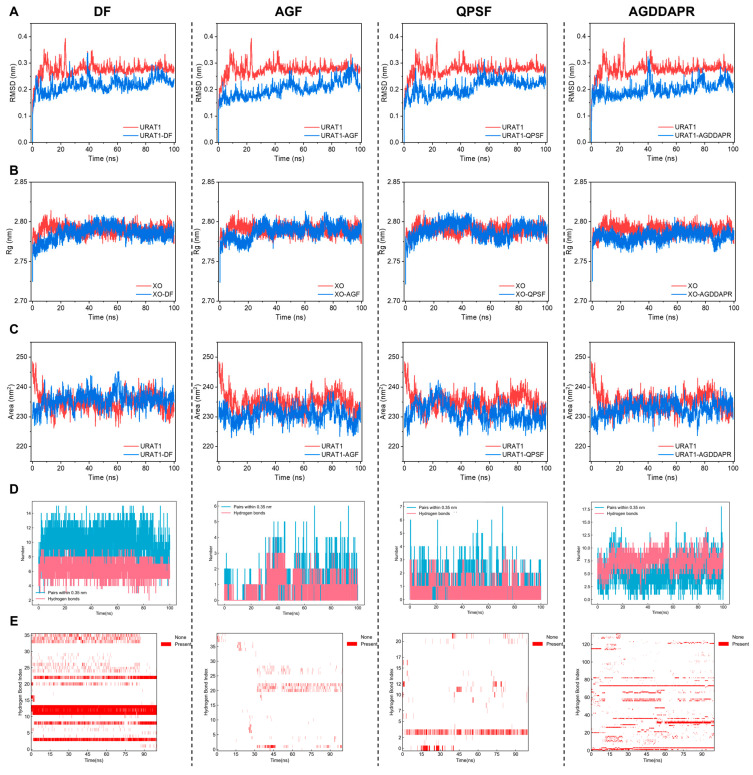
Molecular dynamics analysis of URAT1. (**A**) RMSD, (**B**) Rg, (**C**) SASA, (**D**) the number of hydrogen bonds, and (**E**) hydrogen bond occupancy during the molecular dynamics simulation of DF, AGF, QPSF, and AGDDAPR with URAT1.

Figure 8A presents the root mean square deviation (RMSD) values of URAT1 and the four peptides, namely DF, AGF, QPSF, and AGDDAPR. Notably, the complexes of the peptide DF and peptide AGDDAPR exhibited substantial conformational fluctuations prior to attaining stability during the simulation process. This phenomenon implies that they might potentially interfere with the transport site of URAT1 that is involved in urate transportation, thereby exerting an inhibitory effect on uric acid production. In contrast, the complexes of the peptide AGF and peptide QPSF demonstrated relatively minor fluctuations before reaching stability. Generally, the RMSD values of the four peptide complexes were lower than that of URAT1, and all achieved a steady-state equilibrium. In combination with Figure 8B,C, it was observed that the Rg and SASA values were also relatively lower compared to those of URAT1, indicating that the complexes exhibited enhanced stability in contrast to the single URAT1.

Hydrogen bonds constitute one of the primary driving forces behind the binding of peptides to receptor proteins and play a pivotal role in maintaining a compact and appropriate structure. Figure 8D,E illustrate the number and occupancy of hydrogen bonds, while Figure S2 depicts the angles and distances of the hydrogen bonds with the highest occupancy, along with the amino acid residues involved in their formation and a 2D interaction force diagram of the peptides and URAT1 at the end of 100 ns of simulation. The peptide DF formed 5–10 long-term stable hydrogen bonds with URAT1. Additionally, in conjunction with the molecular docking results and Figure S2, it can be deduced that the peptide DF established multiple hydrogen bonds with the crucial active amino acid residue (Met214) of URAT1 and adjacent amino acid residues (Gly218, Thr219). Moreover, in the 100 ns structure extracted after the completion of the simulation, it engaged in Pi–Pi Stacked and hydrophobic interactions with significant hydrophobic cage amino acid residues (Phe241, Phe449) of URAT1. This might elucidate the mechanism by which the peptide DF can curtail the transport of urate by URAT1 in cells, consequently leading to a reduction in cellular uric acid levels. Although the peptide AGF forms fewer hydrogen bonds with URAT1, it generates four hydrogen bonds with the important amino acid residue Asn39 of URAT1. This might be the reason why it can reduce the amount of uric acid transported by URAT1 at the cellular level. The peptide QPSF forms a relatively small number of hydrogen bonds (0–2) with URAT1. Although the peptide QPSF forms hydrogen bonds with the important amino acid residues (His245, Ser35) of URAT1 during the molecular dynamics process, these bonds are rather unstable during the binding process. This could be the reason why the peptide QPSF fails to reduce the uric acid transported by URAT1 at the cellular level. The peptide AGDDAPR formed 5–10 stable hydrogen bonds with URAT1, including 6 hydrogen bonds formed with several crucial amino acid residues of URAT1 (Arg487, Ser35, and Asn39). Furthermore, in the 100 ns structure obtained after the simulation ended, it engaged in Pi–Pi Stacked and hydrophobic interactions with the important hydrophobic cage amino acid residues (Phe365, Phe241) of URAT1. This might explain the mechanism by which the peptide AGDDAPR can diminish the transport of urate by URAT1 in cells, thereby achieving a reduction in cellular uric acid levels.

#### 3.6.3. GLUT9 Molecular Dynamics Analysis

**Figure 9:**
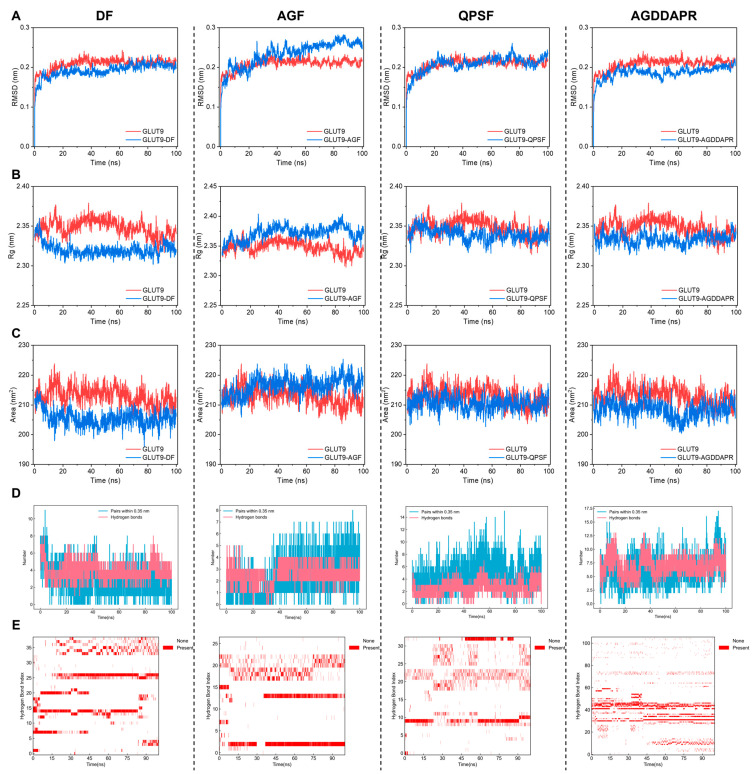
Molecular dynamics analysis of GLUT9. (**A**) RMSD, (**B**) Rg, (**C**) SASA, (**D**) the number of hydrogen bonds, and (**E**) hydrogen bond occupancy during the molecular dynamics simulation of DF, AGF, QPSF, and AGDDAPR with GLUT9.

Figure 9A–C, respectively, present the RMSD (root mean square deviation), Rg (radius of gyration), and SASA (solvent-accessible surface area) values of URAT1 and the four peptides, namely DF, AGF, QPSF, and AGDDAPR. Although the RMSD values of all four complexes reached a stable state, it is evident that the RMSD values of DF-GLUT9 and AGDDAPR-GLU9 were substantially lower than that of the single GLUT9. In contrast, the RMSD values of AGF-GLUT9 and QPSF-GLUT9 were higher than that of the single GLUT9. This clearly suggests that the complexes formed by the peptide DF and peptide AGDDAPRD possess greater stability compared to those of the peptide AGF and peptide QPSF.

Regarding the Rg and SASA values, the complexes of the peptide DF and peptide AGDDAPR were both remarkably lower than those of the single GLUT9. Conversely, the Rg and SASA values of the complex of the peptide AGF were significantly higher than those of the single GLUT9 protein, and the values of the complex of the peptide QPSF were marginally lower than those of the single GLUT9. When considering these RMSD, Rg, and SASA values in combination, it can be inferred that the binding of the peptide DF and peptide AGDDAPR, acting as ligands, to GLUT9 leads to a more stable and compact overall structure.

Hydrogen bonds constitute one of the principal driving forces behind the binding of peptides to receptor proteins and play an essential role in maintaining a compact and appropriate structure. Figure 9D,E depict the number and occupancy of hydrogen bonds, while Figure S3 illustrates the angles and distances of the hydrogen bonds with the highest occupancy, along with the amino acid residues involved in their formation and the 2D interaction force diagram of the peptides and URAT1 at the end of 100 ns of simulation. The peptide DF forms a stable range of 3–6 hydrogen bonds with GLUT9. Notably, among these, the five amino acid residues to which the ten hydrogen bonds with the highest occupancy bind are all key amino acid residues crucial for GLUT9 in transporting urate. This might well be the mechanism through which the peptide DF can effectively diminish the transport of urate by GLUT9 within cells, thereby attaining a reduction in cellular uric acid levels. The peptides AGF and QPSF only manage to form 1–3 stable hydrogen bonds with GLUT9, which could potentially explain their inability to effectively reduce the transport of urate by GLUT9. The peptide AGDDAPR forms 5–10 stable hydrogen bonds with GLUT9, some of which are formed with several important amino acid residues of GLUT9. This may account for the mechanism by which the peptide AGDDAPR can reduce the transport of urate by URAT1 in cells, leading to a decrease in cellular uric acid levels.

## 4. Conclusions

In summary, four peptides (DF, AGF, QPSF, AGDDAPR) with inhibitory activities against hyperuricemia at the cellular level were identified from *Trachurus japonicus* proteins through computer-aided virtual enzymatic hydrolysis, molecular docking, and database screening. Among them, the peptide DF and peptide AGDDAPR affect the generation of hyperuricemia by binding to the active sites of URAT1, GLUT9, and XOD, while the peptide QPSF and peptide AGF mainly influence the generation of hyperuricemia by affecting the active sites of XOD. The results of our molecular dynamics analysis show that forming stable hydrogen bonds with the active sites of target proteins is a key factor in their inhibitory activities against URAT1 and GLUT9. These results further confirm the feasibility and effectiveness of using molecular docking techniques and database screening to identify hyperuricemia-inhibitory peptides. While molecular docking and MD simulations provide valuable insights into the binding interactions between these peptides and the target proteins (XOD, URAT1, and GLUT9), further enzyme kinetic experiments are required to fully validate their inhibitory mechanisms. Future studies will include kinetic assays to determine their inhibition constants (Ki) and explore the potential of these peptides in vivo. This is of crucial value for promoting the application of peptides derived from diverse natural sources in the field of HUA prevention and treatment.

## Figures and Tables

**Table 1:** Peptides predicted for their solubility and toxicity which exhibit binding affinities of ≤−7.0 kcal/mol with three receptor proteins.

Number	Sequence of Peptides	Molecular Weight g/mol	Solubility	Gastrointestinal Absorption	P-Glycoprotein Substrate	Toxin
1	AGF	293.32	High	High	No	None
2	DF	264.28	High	High	No	None
3	QPSF	477.51	High	High	No	None
4	AGDDAPR	700.7	High	High	No	None
5	CPEAL	531.62	High	High	No	None
6	QGVM	433.52	High	Low	Yes	None
7	ADF	351.35	High	Low	No	None
8	AGCSAADSF	827.86	High	Low	Yes	None
9	SGF	309.32	High	High	No	None
10	SAF	323.34	High	Low	No	None
11	CAGAR	476.55	High	Low	Yes	None
12	DGCK	421.47	High	Low	Yes	None
13	SVW	390.43	Extreme high	Low	No	None

## Data Availability

The original contributions presented in this study are included in the article/Supplementary Materials. Further inquiries can be directed to the corresponding author.

## Supplementary Materials

The following supporting information can be downloaded at https://www.mdpi.com/article/10.3390/foods14030524/s1. Figure S1: Molecular docking and dynamics analysis of peptides and XO. Distance, angle, and occupancy of hydrogen bonds and 2D structural diagrams of (A) DF, (B) AGF, (C) QPSF, and (D) AGDDAPR with XO; Figure S2: Molecular docking and dynamics analysis of peptides and URAT1. Distance, angle, and occupancy of hydrogen bonds and 2D structural diagrams of (A) DF, (B) AGF, (C) QPSF, and (D) AGDDAPR with URAT1; Figure S3: Molecular docking and dynamics analysis of peptides and GLUT9. Distance, angle, and occupancy of hydrogen bonds and 2D structural diagrams of (A) DF, (B) AGF, (C) QPSF, and (D) AGDDAPR with GLUT9.
