# Supplementary material for: In Slico Screening and In Vitro Identification of Hyperuricemia-Inhibiting Peptides from Trachurus japonicus
Source: Foods. 2025 Feb 6;14(3):524. doi: 10.3390/foods14030524 (PMC11817512; doi:10.3390/foods14030524)
Supplement: Supplementary file 1 [file foods-14-00524-s001.zip › foods-3430846-supplementary.pdf]

Supporting information

In Slico Screening and In Vitro Identification of Hyperuricemia-Inhibiting Peptides from *Trachurus japonicus*

Zexuan Xu [1,†], Miaoyu Gan [2,†], Weiliang Guan [3], Fang Tian [4], Yuxi Wang [2], Jinjie Zhang [1,*] and Luyun Cai [2,*]

[1] College of Food and Pharmaceutical Sciences, Ningbo University, Ningbo 315211, China; 2211390081@nbu.edu.cn

[2] Ningbo Innovation Center, College of Biosystems and Food Science, Zhejiang University, Ningbo 315100, China; ganmy2021@163.com (M.G.); f265a519@163.com (Y.W.)

[3] School of Light Industry and Food Engineering, Guangxi University, Nanning 530004, China; wlguan@gxu.edu.cn

[4] Key Laboratory of Health Risk Factors for Seafood of Zhejiang Province, School of Food and Pharmacy, Zhejiang Ocean University, Zhoushan 316022, China; tianfang@zjou.edu.cn

* Correspondence: jackace@163.com (J.Z.); cailuyun@zju.edu.cn (L.C.)

† These authors contributed equally to this work.

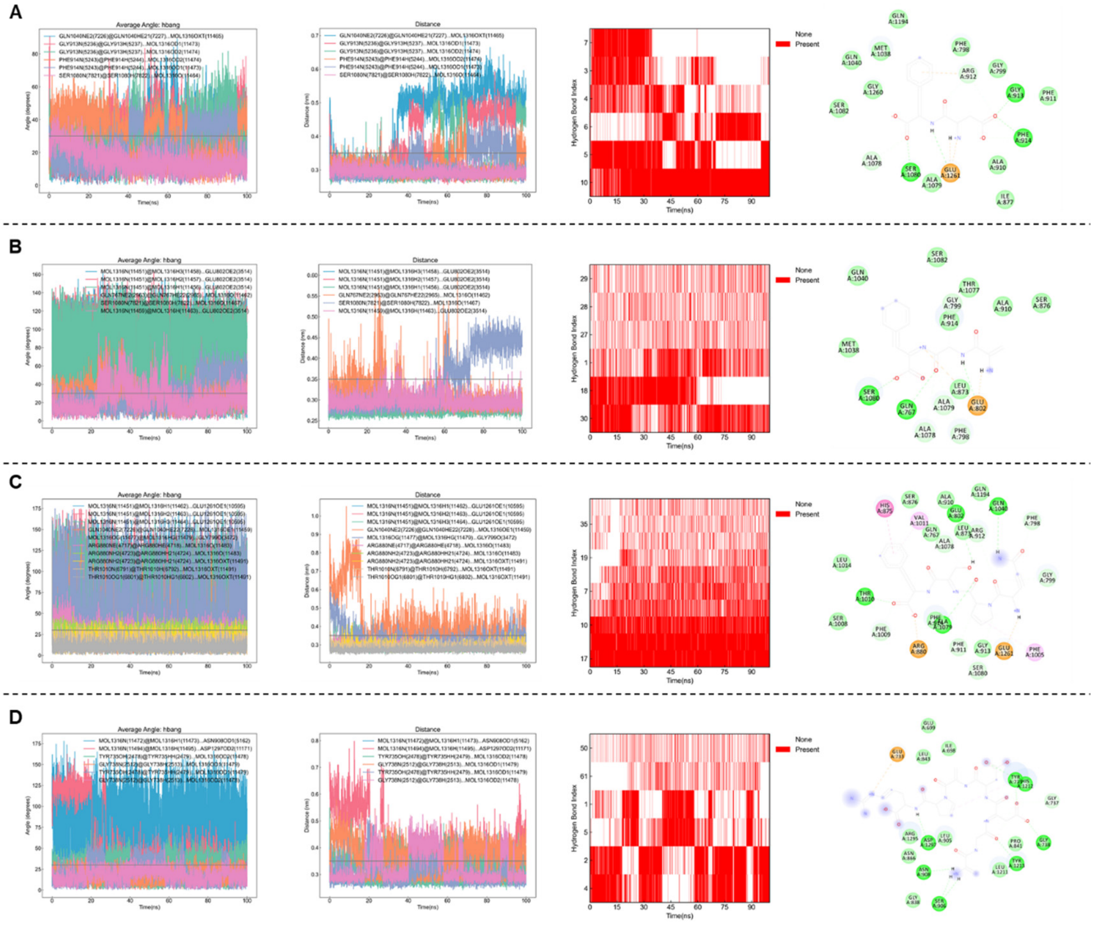

Figure S1. Molecular docking and dynamics analysis of peptides and XO. Distance, angle, and occupancy of hydrogen bonds and 2D structural diagrams for (A) DF, (B) AGF, (C) QPSF, and (D) AGDDAPR with XO.

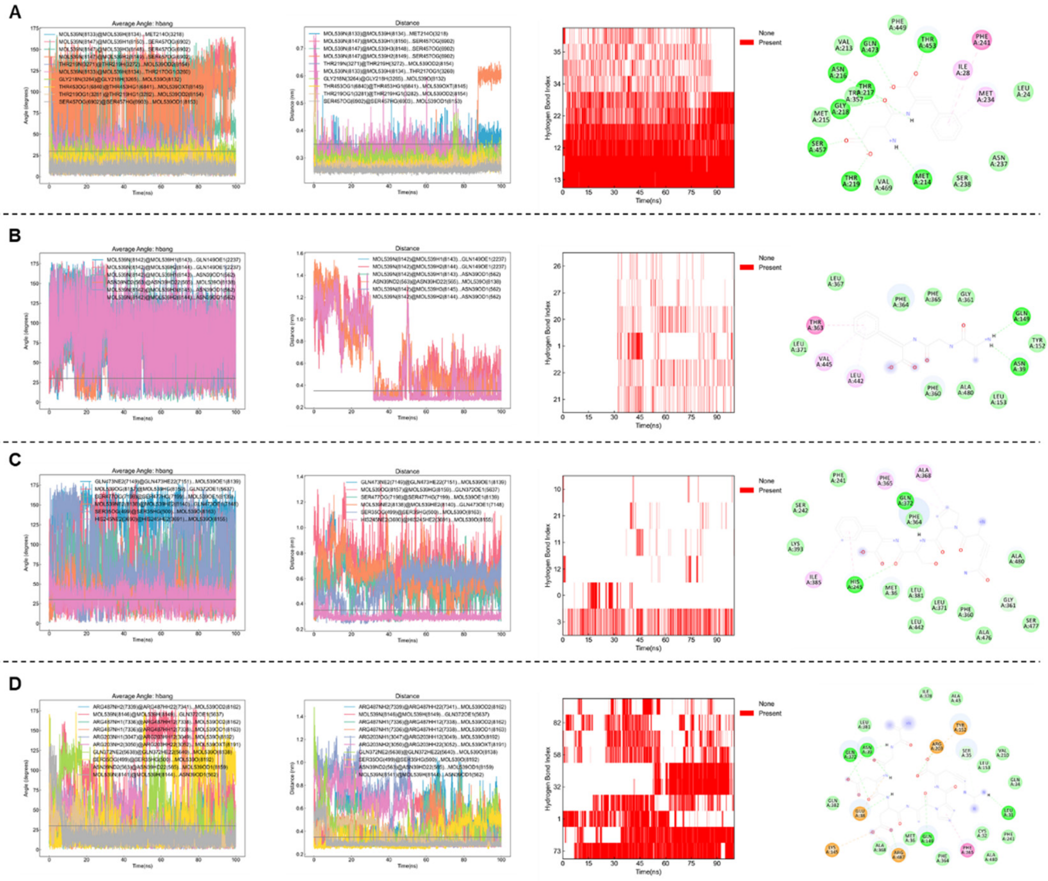

Figure S2. Molecular docking and dynamics analysis of peptides and URAT1. Distance, angle, and occupancy of hydrogen bonds and 2D structural diagrams for (A) DF, (B) AGF, (C) QPSF, and (D) AGDDAPR with URAT1.

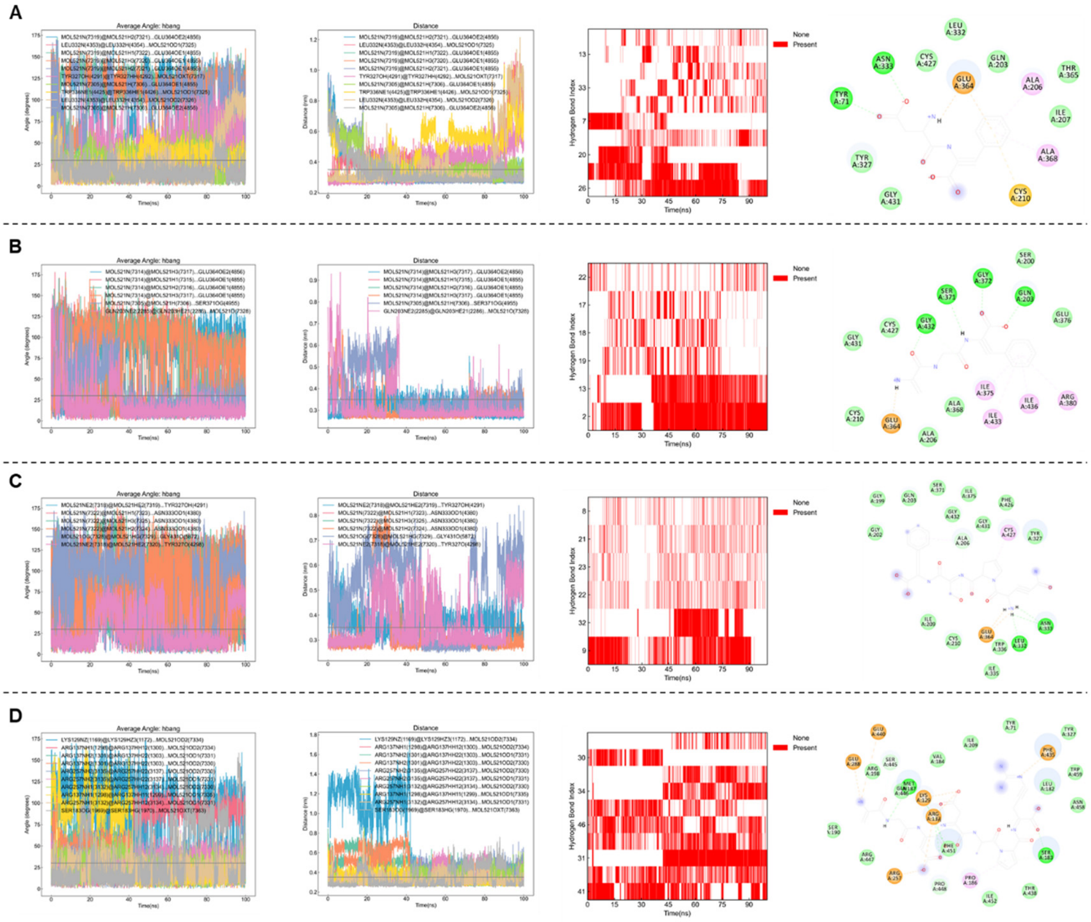

Figure S3. Molecular docking and dynamics analysis of peptides and GLUT9. Distance, angle, and occupancy of hydrogen bonds and 2D structural diagrams for (A) DF, (B) AGF, (C) QPSF, and (D) AGDDAPR with GLUT9.
